# Gene expression microarray public dataset reanalysis in chronic obstructive pulmonary disease

**DOI:** 10.1371/journal.pone.0224750

**Published:** 2019-11-15

**Authors:** Lavida R. K. Rogers, Madison Verlinde, George I. Mias

**Affiliations:** 1 Microbiology and Molecular Genetics, Michigan State University, East Lansing, MI, United States of America; 2 Institute for Quantitative Health Science and Engineering, Michigan State University, East Lansing, MI, United States of America; 3 Biochemistry and Molecular Biology, Michigan State University, East Lansing, MI, United States of America; University of Alabama-Birmingham, UNITED STATES

## Abstract

Chronic obstructive pulmonary disease (COPD) was classified by the Centers for Disease Control and Prevention in 2014 as the 3^rd^ leading cause of death in the United States (US). The main cause of COPD is exposure to tobacco smoke and air pollutants. Problems associated with COPD include under-diagnosis of the disease and an increase in the number of smokers worldwide. The goal of our study is to identify disease variability in the gene expression profiles of COPD subjects compared to controls, by reanalyzing pre-existing, publicly available microarray expression datasets. Our inclusion criteria for microarray datasets selected for smoking status, age and sex of blood donors reported. Our datasets used Affymetrix, Agilent microarray platforms (7 datasets, 1,262 samples). We re-analyzed the curated raw microarray expression data using R packages, and used Box-Cox power transformations to normalize datasets. To identify significant differentially expressed genes we used generalized least squares models with disease state, age, sex, smoking status and study as effects that also included binary interactions, followed by likelihood ratio tests (LRT). We found 3,315 statistically significant (Storey-adjusted q-value <0.05) differentially expressed genes with respect to disease state (COPD or control). We further filtered these genes for biological effect using results from LRT q-value <0.05 and model estimates’ 10% two-tailed quantiles of mean differences between COPD and control), to identify 679 genes. Through analysis of disease, sex, age, and also smoking status and disease interactions we identified differentially expressed genes involved in a variety of immune responses and cell processes in COPD. We also trained a logistic regression model using the common array genes as features, which enabled prediction of disease status with 81.7% accuracy. Our results give potential for improving the diagnosis of COPD through blood and highlight novel gene expression disease signatures.

## Introduction

Chronic obstructive pulmonary disease (COPD) impairs lung function and reduces lung capacity. In COPD there is inflammation of the bronchial tubes (chronic bronchitis) [1] and destruction of the air sacs (emphysema) [2] within the lungs [3–6]. Chronic bronchitis and emphysema often occur together and are grouped under COPD [1, 2]. Furthermore, the Global Initiative for Chronic Obstructive Lung Disease (GOLD) describes COPD as a common and preventable disease that is caused by exposure to harmful particles and gases that affect the airways and alveolar of the lungs [7, 8]. Individuals with COPD experience shortness of breath due to lowered concentrations of oxygen in the blood and a chronic cough accompanied by mucus production [1–4, 6]. COPD progresses with time and the damage caused to the lungs is irreversible [8, 9], and we do not currently have adequate therapies to control COPD progression.

COPD, the 3^rd^ leading cause of death in the United States (US), is expected to rise in 15 years to the leading cause of death [8–10]. Globally, there were over 250 million cases of COPD reported in 2016 and in 2015 3.17 million individuals died from the disease [5]. COPD is prevalent in low- and middle-income countries with over 90% of COPD cases occurring in these areas [5, 10]. The disease is mainly caused by tobacco exposure through smoking cigarettes or second-hand exposure to smoke [8, 9]. In addition to this, continuous exposure to other irritants such as burning fuels, chemicals, polluted air and dust can lead to COPD [5]. Cigarette smoke exposes the lungs to large amounts of oxidants that induce inflammation of the airways. Studies have also suggested that COPD acts like an autoimmune disease due to persistent inflammation even after smoking has ceased [11–13]. In addition to environmental pollutants, there is also also a genetic deficiency, alpha-1 antitrypsin deficiency (AATD), that is associated with COPD [8]. AATD protects the lungs, and without it the lungs become vulnerable to COPD. The prevalence of COPD is expected to rise due to increasing smoking rates and larger populations of elderly individuals in many countries [5].

COPD is often underdiagnosed and despite tobacco exposure being the highest risk factor, not all smokers get COPD, and non-smokers can also develop COPD. Previous work has been done to identify biomarkers for earlier diagnosis of COPD in blood, a non-invasive approach. Bahr et al., compared expression profiles of smokers with COPD and smokers without COPD [14]. They used multiple linear regression to identify candidate genes and pathways. Their results highlighted pathways involved in the immune system and inflammatory response [14]. Another study of blood gene expression in COPD explored using pre-existing gene interaction networks to perform unsupervised clustering to identify COPD disease sub-types [15]. More recently, Reinhold et al., took a different approach by conducting a meta-analysis that identified groups of genes associated with COPD by using consensus modules of gene co-expression. They built networks of genes that were co-expressed and associated with COPD phenotypes [16].

**Fig 1:**
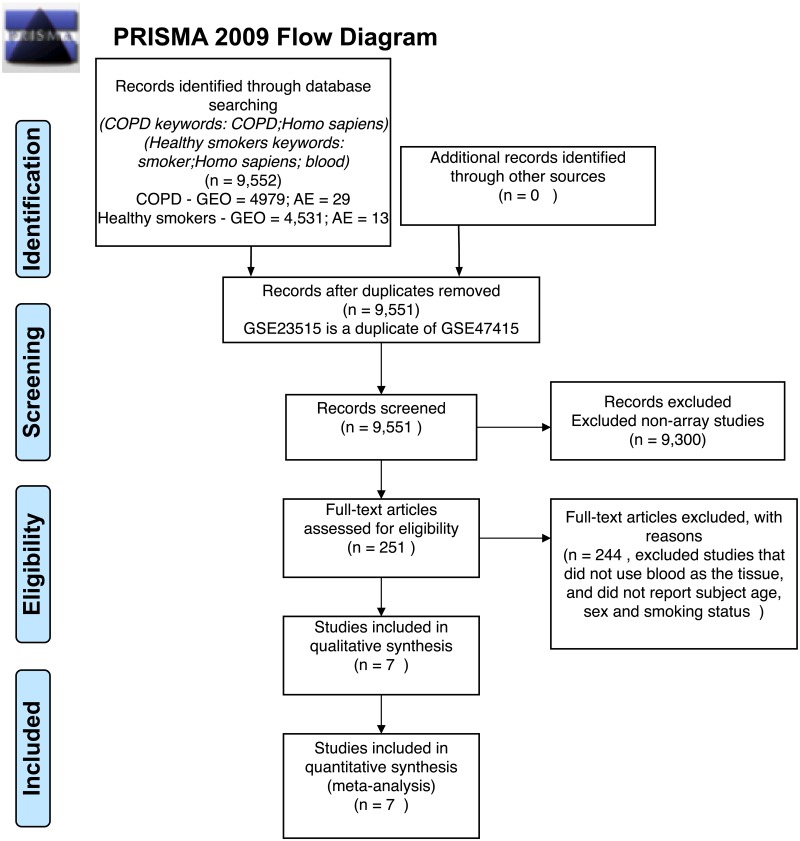
Preferred Reporting Items for Systematic Reviews and Meta-Analyses (PRISMA) flow diagram. Data were curated from Gene Expression Omnibus (GEO) and Array Express (AE). The PRISMA flow diagram shows the identification, screening, eligibility and inclusion of samples in our analysis.

**Fig 2:**
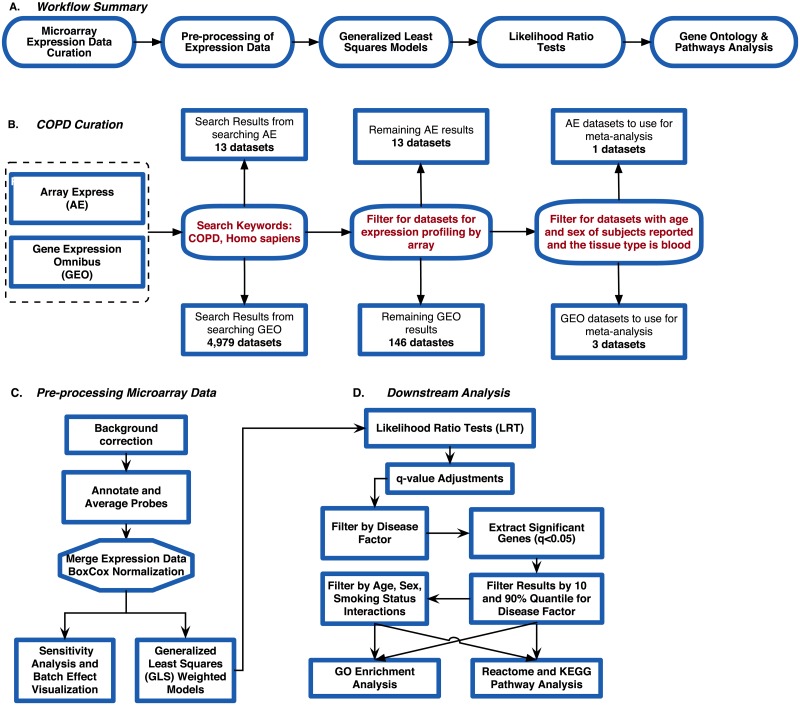
Analysis pipeline for chronic obstructive pulmonary disease. (A)Summary of workflow used for the re-analysis of microarrays, (B) Pre-processing steps used on the microarray data, (C) Data analysis post generalized least squares (GLS) model fit, (D)downstream analysis steps using GLS.

In our reanalysis, the objective was to identify the effects of age, sex, and smoking status on gene expression in COPD. We investigated gene expression changes in blood for 1,262 samples (574 healthy samples and 688 COPD samples) to identify genes and their associated pathways in COPD (Figs 1 and 2, S1 and S2 Files—see also Methods below). Our study is the largest reanalysis of public microarray datasets on blood expression for COPD to date, to the best of our knowledge, and our results offer prospective gene and pathway associations that may be targeted for improving COPD diagnosis and treatment. Our analysis also highlighted disease genes that interact with smoking status, and these genes can be used to further characterize the effects of smoking on COPD development.

## Materials and methods

We used seven publicly available COPD microarray gene expression datasets in our reanalysis of microarray data to evaluate variation in gene expression across samples due to disease status, sex, age and smoking status (Table 1). The 7 expression datasets were from 3 different microarray platforms: Affymetrix GeneChip Human Genome U133 Plus 2.0, Affymetrix Human Gene 1.1 ST Array and Agilent Whole Human Genome Microarray 4x44K. Our current reanalysis pipeline (similar to Brooks et al. [17]), included 4 main steps (Fig 2): (1) data curation; (2) pre-processing of raw expression data; (3) Generalized least squares (GLS) weighted models (accounting for variance heterogeneity) which compared gene expression changes due to disease state, smoking status, sex and age group; (4) likelihood ratio tests (LRT) determination of differential expression for multiple factors and filtering for biological significance, followed by Gene ontology (GO) and pathway enrichment analysis of the differentially expressed and biologically significant genes.

**Table 1:** Description of datasets used in the reanalysis.

Database Repository	Dataset Accession	Control	COPD	Platform
Array Express	E-MTAB-5278	181	53	Affymetrix Human Genome Plus 2.0
Array Express	E-MTAB-5279	89	0	Affymetrix Human Genome Plus 2.0
GEO	GSE42057	42	94	Affymetrix Human Genome Plus 2.0
GEO	GSE47415	48	0	Agilent-014850 Whole Human Genome Microarray 4x44K
GEO	GSE54837	90	136	Affymetrix Human Genome Plus 2.0
GEO	GSE71220	44	405	Affymetrix Human Gene 1.1 ST Array
GEO	GSE87072	80	0	Affymetrix Human Genome Plus 2.0

### Microarray data curation from Gene Expression Omnibus and Array Express

To gather the datasets for our reanalysis, we searched the National Center for Biotechnology Information (NCBI)’s data repository, Gene Expression Omnibus (GEO) [18], and the European Bioinformatics Institute (EMBL-EBI)’s data repository, Array Express (AE) [19] for microarray expression data. We used the following keywords to search the repositories: COPD, *Homo sapiens*, blood (whole blood and peripheral blood mononuclear cells) and expression profiling by array (Fig 1). The search results were further filtered to include datasets where the age, sex and smoking status of the samples were reported (Fig 1). We found 3 datasets from GEO (GSE42057 [20], GSE71220 [21], GSE54837 [22]) and 1 from AE (E-MTAB-5278 [23]) that met our search criteria (Table 1 and Fig 1). We conducted an additional search on GEO and AE to find healthy subjects with their smoking history reported to balance our control subjects with our COPD subjects. The search keywords included: *Homo sapiens*, blood, smoking and expression profiling by array. We also filtered these search results for datasets that reported the age, sex and smoking status of subjects. With this additional search, we added 3 more datasets: GSE87072 [24], GSE47415 [25], and E-MTAB-5279 [23] which helped improve the balance between COPD and control subjects (Table 1 and DF1 of online data files, see also S3 File for online data files guide).

After selecting the datasets for our analysis, we retrieved the raw microarray expression data for each dataset, and created a demographics file per study, which included sample characteristics using e-utils in Mathematica [26] (Table 2). The demographics files were further filtered to eliminate samples that did not fit our inclusion criteria. For example, GSE71220 included subjects that were using statin drugs [21], and hence we excluded all samples that were receiving treatment from our analysis. For GSE87072, we removed the samples that were moist snuff consumers [24] and only used smokers and non-smokers in our analysis. In our additional search for controls with smoking status reported, we filtered the selected datasets (GSE87072, GSE47415 and E-MTAB-5279) and only used the healthy samples for our analysis. In addition to this, we excluded the subjects in GSE23515 [27] from our analysis because 22 of the 24 samples are duplicates from GSE47415 [25]. Our demographics files were created to include variables that were reported across all samples (see merged Demographics file DF1 of online supplementary data files) because study annotations had not been uniformly reported in the databases (S2 File).

**Table 2:** Sample characteristics by dataset.

Dataset Accession	Sex(M/F)	Smoking Status (S/NS/FS)*	Age Range
E-MTAB-5279	46/43	30/29/30	24–65
EMTAB5278	136/98	114/60/60	41–70
GSE42057	74/62	35/2/99	45–80
GSE47415	24/24	24/24/0	20–64
GSE54837	148/78	84/6/136	40–75
GSE71220	285/165	91/22/336	49–75
GSE87072	80/0	40/40/0	35–60

*S = smoker, NS = non-smoker, FS = former smoker

### Microarray pre-processing and BoxCox normalization

To download the raw microarray expression for each dataset we used Mathematica [28]. All raw expression data files were pre-processed in R [29] using R packages specific to each microarray platform (Fig 2B). For the datasets from the Affymetrix Human Genome Plus 2.0 platform, we used the affy package [30] for pre-processing all of the .CEL files. The oligo [31] and affycoretools [32] packages were used to pre-process the data files from the Affymetrix Human Gene 1.1 ST microarry platform, while the limma package [33] was used for the data files from the Agilent Whole Human Genome microarray platform. We performed background correction, normalization, and all probes were annotated and summarized (Fig 2B). For the Affymetrix Human Genome Plus 2.0 expression data files, the expresso function was used to pre-process the files with the following parameters: background correction with robust multi-array analysis (RMA), correcting the perfect-match (PM) probes, and ‘avdiff’ to calculate expression values [30]. Subsequently, the avereps function from limma was used to summarize the probes and remove replicates [33]. The Affymetrix Human Gene 1.1 ST data files were also background corrected using RMA, and the probes were summarized and replicates removed using the avereps function. As for the Agilent data files, background correction was performed using the backgroundCorrect function with NormExp Background Correction as the method from the limma package [34]. The probes for both Affymetrix Human Gene 1.1 ST and Agilent were also summarized and replicates were removed using the avereps function from limma. Once pre-processing was completed, the 8 datasets (Table 1) were merged by common gene symbols into a single matrix file. Using the ApplyBoxCoxTransform function and the StandardizeExtended function from the MathIOmica (version 1.1.3) package [26, 35] in Mathematica, we performed a Box-Cox power transformation and data standardization on the merged expression file [36] (Fig 2B and DF2 of online supplementary data files).

### Sensitivity analysis: Identifying and visualizing batch effects

**Fig 3:**
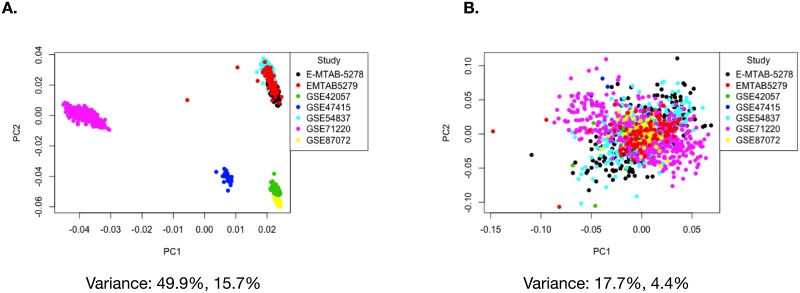
Visualizing batch effects introduced by using multiple studies in our analysis. (A) PCA before and (B) PCA after batch effect correction with ComBat.

**Fig 4:**
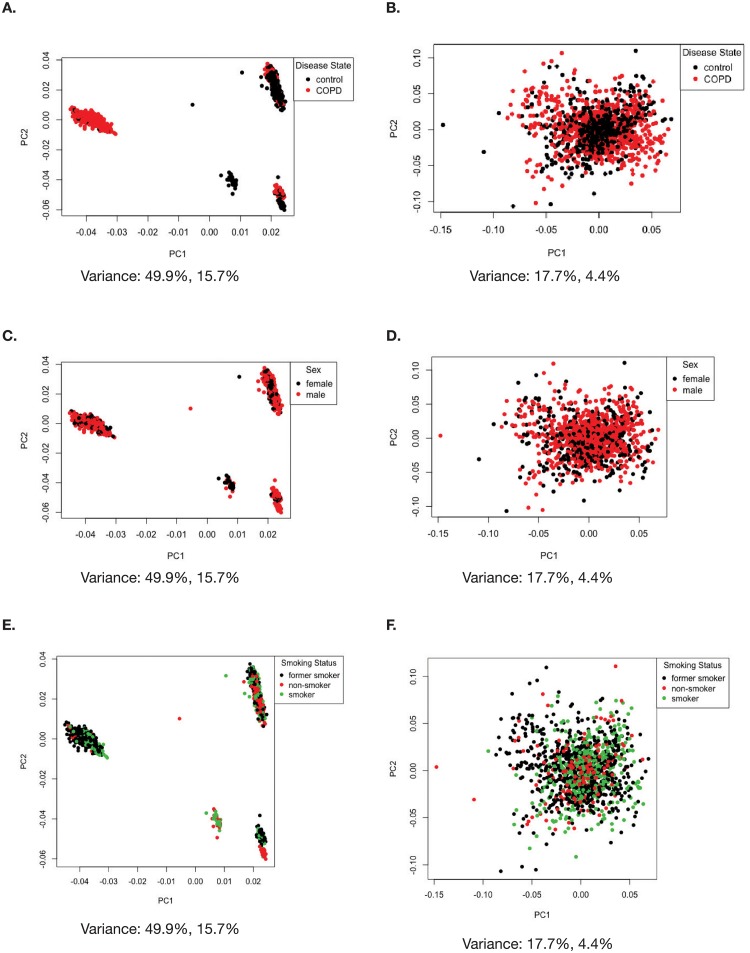
Visualizing batch effects introduced by using multiple studies by looking at the factors disease state, sex and smoking status in our re-analysis. (A) PCA before (disease factor), (B) PCA after batch effect correction with ComBat (disease factor), (C) PCA before (sex) (D) PCA after batch effect correction with ComBat (sex), (E) PCA before (smoking status) (F) PCA after batch effect correction with ComBat (smoking status).

Conducting reanalysis by combining expression datasets across different microarray platforms and research labs/studies introduces batch effects/confounding factors to the data. The batch effects can introduce non-biological variation in the data, which affects the interpretation of the results. In order to determine and visualize potential variation in the expression data across factors, we conducted principal component analysis (PCA) on the expression data and generated PCA plots (Figs 3 and 4). As we also previously described [17], the study factor is directly related to the microarray platform type. To address this, the ComBat function in the sva package was used to correct for variation in the data due to the study factor [37, 38] (see also DF3 of online supplementary data files). PCA plots were used to visualize variation in expression data before and after batch correction with ComBat [39] (Figs 3 and 4), confirming the main batch effect removal by adjusting for study, and also illustrating the unequal variances within study groups.

### Using GLS models to identify differentially expressed genes by factor

We tested the data per gene and determined that variances were heterogeneous in more than 10% of the data (using Brown-Forsythe tests [40], implemented using the levene.test of the lawstat package [41]). We subsequently implemented a GLS model, for each gene’s expression data, also adding weights to account for differences in variances within studies. To determine if the factors of disease status, sex, study, and smoking status had an impact on gene expression in COPD, we modeled (see GLS model below) our merged expression matrix (DF2 of online supplementary data files) and then conducted LRT to identify differentially expressed genes (Fig 2B). Schematically our full GLS formula for gene expression, *g*, per each gene included main effects and interactions:

$$\begin{array}{c} g∼\;\sum_{i}x_{i}+\sum_{i,j;j>i}x_{i}:x_{j}+study \end{array}$$ (1)

where *x*_*i*_∈ {age group, sex, smoker, disease status} and the factors have the following levels:

- disease status = {control, COPD}
- sex = {male, female}
- age group = {under 50, 50-55, 55-60, 60-65, 65-70, over 70}
- smoker = {non-smoker, former smoker, smoker}
- study = {GSE42057, GSE47415, GSE54837, GSE71220, GSE87072, E-MTAB-5278, E-MTAB-5279}

The factors and interactions were tested for marginal effects by fitting the appropriate model with the factors included/removed respectively in the LRT model marginal effect tests. The GLS model was implemented using the nlme package [42]. False discovery rates (FDR) were controlled for multiple testing using Storey q-values [43], using package qvalue [44] (see also DF4-DF5 of online supplementary data files for q-values and model estimates). Genes were considered statistically significant if their q-values were <0.05. We focused on the GLS results for the disease factor, and filtered them for q-values <0.05, as well as interactions between disease and sex, and disease and smoking (see below, and also DF5-DF8 of online supplementary data files). These filtered genes were then identified as statistically significant disease genes. We used this gene list to identify what GO terms and Kyoto Encyclopedia of Genes and Genomes (KEGG) and Reactome pathways they were enriched in. We used the GOAnalysis and KEGGAnalysis functions from the MathIOmica package for GO and KEGG pathway enrichment respectively. Additionally, we used the enrichPathway function from the ReactomePA package in R [45]. All functions for enrichment analysis used the BH p-value correction method and GO terms, KEGG and Reactome pathways with a BH-adjusted p-value <0.05 were considered statistically significant (see DF9-DF10 of online supplementary data files for full MathIOmica output).

To determine the biological effect of the LRT statistically significant genes (disease status factor) and calculate relative expression (difference in means) to determine up- or down- regulation of genes, we used GLS estimates to assess the top changing genes by biological effect, using the two-tailed 10 and 90% quantiles. With these results we carried out GO and pathway enrichment to identify which biological processes and pathways the genes were enriched. All GO terms and pathways with a Benjamini-Hochberg (BH)-adjusted p-value <0.05 were considered significant [46].

### Machine learning with COPD

Machine learning classification was carried out in Mathematica using the Classify function [47], with the Method parameter set to “LogisticRegression”. We first trained on all 1262 samples, using all the common gene expression data estimates as features. We also randomized the dataset, and created 10 sets for training and testing, with 90% of the samples used for training, and 10% of the samples used for testing, where the 10 testing sets were mutually exclusive (10-fold cross-validation).

## Results

Our re-analysis selection criteria for data curation (Fig 1) resulted in 8 datasets from GEO and AE (Table 1). After pre-processing the data, we combined all datasets into a large matrix by merging by common gene names. This data merge resulted in 1,262 samples (574 controls and 688 COPD subjects) and 16,237 genes. Our 1,262 samples consists of 792 males and 470 females, and also 661 former smokers, 418 current smokers and 183 non-smokers.

### Sensitivity analysis and batch effects

Prior to designing our linear model, we wanted to visualize variation introduced into the data due to batch effects, and how the variation changes when the data is adjusted with ComBat for batch effects. We used ComBat in R to adjust for the study effect on the data and generated PCA plots before and after batch correction (Fig 3). In Fig 3A, before running ComBat, the data separates into four major clusters with a variance of 49.9% in PC1 and 15.7% in PC2. After running ComBat, the clustering of the data is removed, and variance reduced to 17.7% in PC1 and 4.4% in PC2 (Fig 3B). We also plotted the PCAs for the other factors (Fig 4) before and after using ComBat for batch effect correction. The ComBat batch effect corrected expression data was only used to assess and visualize changes in variation due to removal of batch and to confirm the inclusion of study as an effect factor in our linear model.

### Differences in gene expression due to disease status

**Fig 5:**
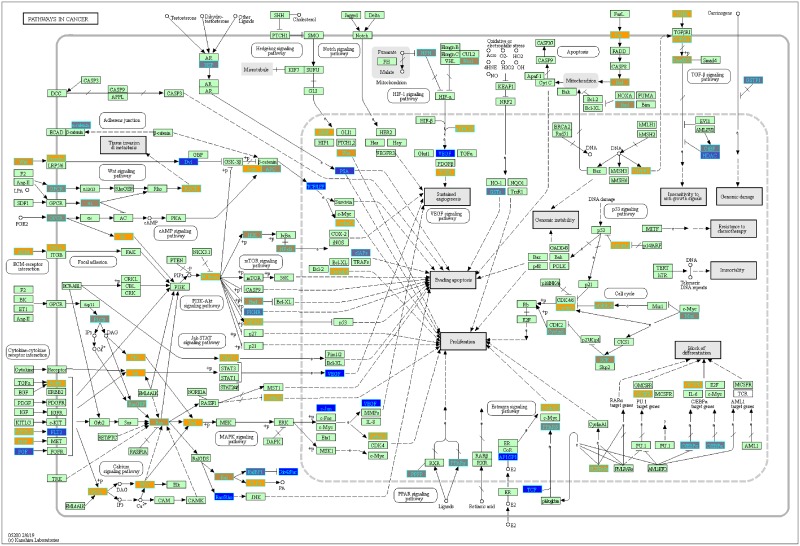
Highlighted pathways in cancer (hsa5200) with enriched genes from the LRT (q-value < 0.05) [49–51]. Yellow-colored genes are up-regulated and blue-colored genes are down-regulated in COPD samples.

**Fig 6:**
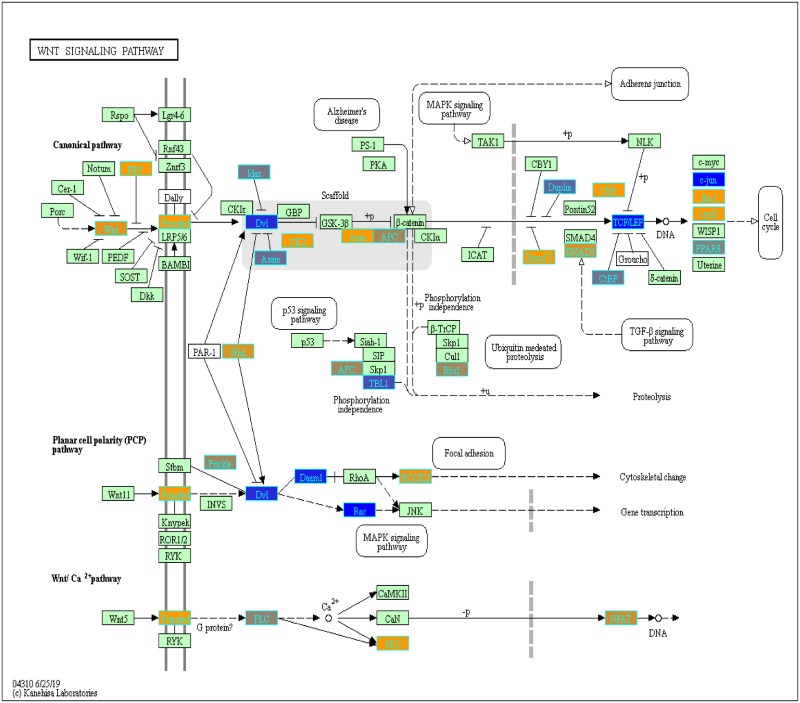
Highlighted Wnt signaling KEGG pathway (hsa04310) with enriched genes from the LRT (q-value < 0.05) [49–51]. Yellow-colored genes are up-regulated and blue-colored genes are down-regulated in COPD samples.

**Fig 7:**
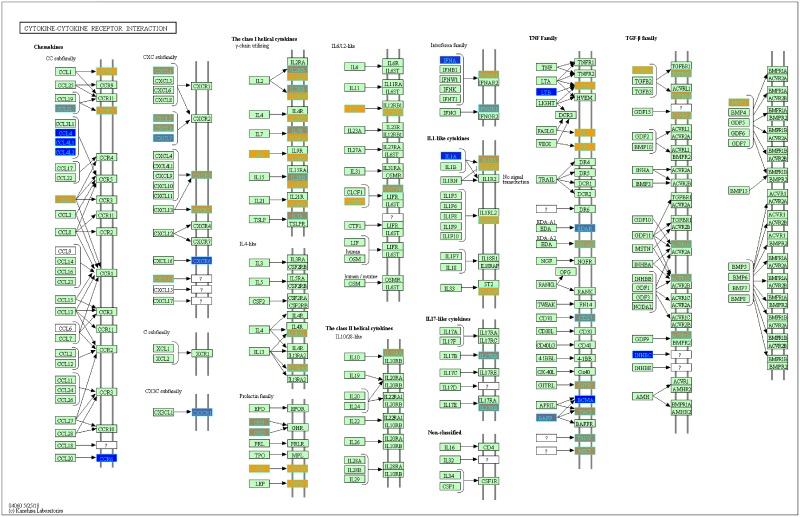
Highlighted Cytokine-cytokine receptor interaction KEGG pathway (hsa04060)with enriched genes from the LRT (q-value < 0.05) [49–51]. Yellow-colored genes are up-regulated and blue-colored genes are down-regulated in COPD samples.

**Fig 8:**
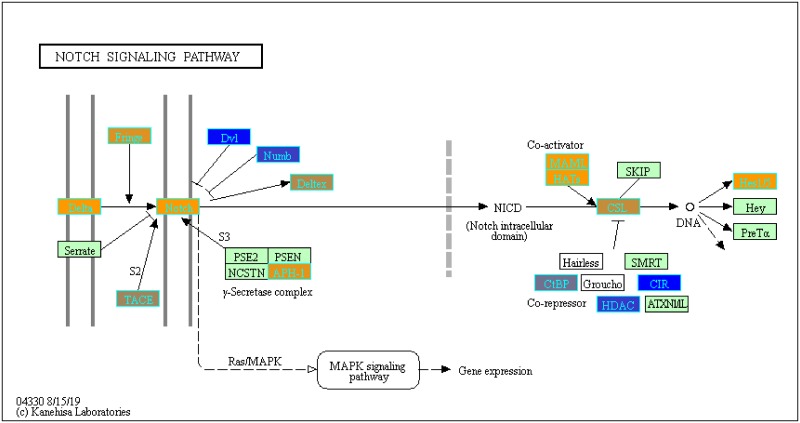
Highlighted notch signaling KEGG pathway (hsa04330)with enriched genes from the LRT (q-value < 0.05) [49–51]. Yellow-colored genes are up-regulated and blue-colored genes are down-regulated in COPD samples.

With our LRT results, we were able to evaluate variance in gene expression introduced by each factor and their pair-wise interactions (c.f. Pavlidis et al. [48]). To determine which genes from our LRT results were statistically significant by the disease status factor, we filtered the genes by using q-value <0.05. We found 3,315 statistically significant disease genes (see DF4 of online supplementary data files). We performed GO and pathway enrichment analysis on the 3,315 genes. Our enriched GO terms included: protein binding (1636), transcription (319 gene hits), innate immune response (66 gene hits), inflammatory response (69 gene hits), adaptive immune response (29 gene hits), apoptotic process (94 gene hits), response to drug (75 gene hits) (see DF10 of online supplementary data files for full table). We found 26 enriched KEGG pathways (Table 3 and DF9 of online supplementary data files). The enriched KEGG pathway analysis results include: Pathways in cancer (89 gene hits, Fig 5), Wnt signaling (40 gene hits, Fig 6), Cytokine-cytokine receptor interaction (61 gene hits, Fig 7), and Notch signaling (18 gene hits, Fig 8)—see also Table 3 and DF11-DF14 of online supplementary data files. We used the KEGGPathwayVisual function in the MathIOmica package to highlight whether our gene hits for the top 4 enriched KEGG pathways were up- or down- regulated in the pathway (based on GLS estimates following two-tailed filter (10% and 90% quantiles), Figs 5–8. For example, Fig 5 depicts the Pathways in Cancer KEGG pathway and highlights our gene hits (with yellow: up-regulated, and blue: down-regulated gene expression). In this pathway, Fig 5, our results indicate that genes such as TGF-β which is involved in insensitivity to anti-growth signals as well as CyclinD1(role in cell proliferation) are both up-regulated in COPD compared to controls.

**Table 3:** Enriched top 10 KEGG pathways using the differentially expressed genes from disease factor.

KEGG ID	KEGG Pathway Name	Gene Count	*p-value*	*adjusted p-value*
path:hsa05200	Pathways in cancer	89	3.3734E-07	0.00010019
path:hsa04310	Wnt signaling pathway	40	2.88289E-06	0.000428109
path:hsa04060	Cytokine-cytokine receptor interaction	61	1.07931E-05	0.001068519
path:hsa04330	Notch signaling pathway	18	2.41938E-05	0.001796392
path:hsa04151	PI3K-Akt signaling pathway	71	8.28419E-05	0.004920808
path:hsa04810	Regulation of actin cytoskeleton	49	0.000103424	0.005119501
path:hsa04010	MAPK signaling pathway	55	0.00018911	0.007967647
path:hsa01210	2-Oxocarboxylic acid metabolism	9	0.000215638	0.007967647
path:hsa04014	Ras signaling pathway	50	0.000241444	0.007967647
path:hsa04152	AMPK signaling pathway	31	0.000413929	0.011956858

**Fig 9:**
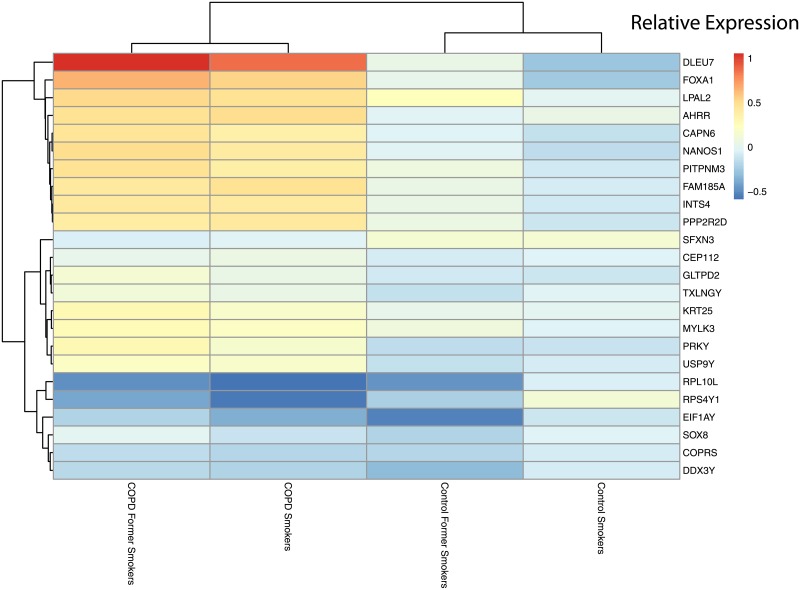
Heatmap of statistically significant interacting genes across disease states and smoking statuses. Relative expression calculated using control non-smokers as the baseline.

Of the 3,315 disease genes we further filtered our LRT results (see DF4 of online supplementary data files) to identify genes with statistically significant interactions with smoking status (disease:smoking status, adjusted q-values < 0.05). We found 24 genes that had a statistically significant pairwise interaction between disease status and smoking status (see DF8 of online supplementary data files). Using the 24 interacting genes, we calculated the relative expression across the different pairings of smoking status and disease status to compare expression (Fig 9). We used the GLS estimates’ differences of the non-smoking controls as our baseline to calculate the difference in means for the different disease and smoking groups. In Fig 9 the data cluster by disease state (COPD together and controls together), and smokers and former smokers across both disease states have similar expression profiles. There are subset of genes that are over expressed in COPD smokers compared to control non-smokers as well as a subset of genes that are down-regulated. Finally, control smokers and former smokers have similar expression profiles with GGT6 being an outlier (Fig 9).

### Up and down- regulated gene expression in COPD

To assess biological effect and determine factorial differences in gene expression we conducted LRT on our GLS models, and used estimates to measure effect size on our 3,315 statistically significant disease genes. We first focused on COPD and control gene expression differences and used q-value <0.05 to determine significance. We also filtered further by using a 10% two-tailed quantile cutoff to identify significantly up- and down- regulated genes (DF15-DF17 of online supplementary data files). Once we filtered by q-value, we calculated to 10 and 90% quantiles using estimates. For the COPD-control LRT comparisons we found 679 statistically significant genes that we classified as down-regulated (mean differences ⪅-0.2201) and up-regulated (mean differences ⪆0.4043) in our COPD subjects. Of the 679 differentially expressed genes (DEG), 280 genes were down-regulated and 399 genes were up-regulated (DF16-DF17) of online supplementary data files). The top 25 up- and down- regulated genes are displayed in Table 4. KEGG enrichment analysis on the 280 down-regulated disease genes resulted in two significantly enriched pathways: Ribosome (12 Gene hits) and Non-alcoholic fatty acid liver disease (8 gene hits).

**Table 4:** Top 25 up and down regulated differentially expressed genes in COPD based on effect size.

Up-Regulated			Down-Regulated		
Gene	Difference of Means	adjusted q-value	Gene	Difference of Means	adjusted q-value
DUSP7	0.545	6.9E-06	RPS4Y1	-0.441	5.7E-13
GPR15	1.020	2.7E-05	FCGR1B	-0.610	6.9E-05
PLD1	0.411	8.0E-05	LOC93622	-0.637	1.1E-04
FICD	0.417	1.0E-04	TCF7	-0.251	3.1E-04
CBLL1	0.460	3.1E-04	NFIL3	-0.234	5.5E-04
SMURF1	0.513	3.7E-04	COPB2	-0.256	8.9E-04
HIST1H3I	1.242	4.2E-04	RAB13	-0.293	1.3E-03
MSL2	0.449	4.5E-04	MYOM2	-0.672	1.3E-03
FAM185A	0.446	5.8E-04	CLEC5A	-0.321	1.3E-03
PPP2R2D	0.424	6.4E-04	LINC01138	-0.288	1.3E-03
EPHB1	0.421	8.1E-04	FBRSL1	-0.236	1.3E-03
MR1	0.460	9.5E-04	NACA	-0.276	1.4E-03
AHRR	0.467	9.6E-04	ZBTB4	-0.317	2.0E-03
TCEANC2	0.681	1.1E-03	NMI	-0.268	2.1E-03
GPR141	0.976	1.1E-03	TXNDC17	-0.721	2.1E-03
YKT6	0.420	1.2E-03	HSBP1	-0.435	2.5E-03
CRNN	0.515	1.2E-03	CD163	-0.632	2.6E-03
FOXA1	0.727	1.4E-03	RPL10L	-0.366	2.8E-03
TNRC6C	0.414	1.6E-03	ATP6V1D	-0.232	3.1E-03
TCF12	0.423	1.7E-03	SETD1B	-0.266	3.1E-03
UBXN7	0.413	1.7E-03	CBR3	-0.223	3.2E-03
SORT1	0.424	1.7E-03	HIP1R	-0.252	3.6E-03
ATG7	0.437	1.8E-03	PROS1	-0.246	3.8E-03
TEC	0.457	1.9E-03	CHMP5	-0.248	4.3E-03
TMLHE	0.505	2.0E-03	MMEL1	-0.416	4.4E-03

As for the 399 up-regulated genes, the KEGG pathway Jak-STAT signaling pathway was enriched with 10 gene hits. We also wanted to compare our gene list to a previously published meta-analysis. Reinhold et al., had a total of 6,243 genes which they grouped into 15 modules for each cohort [16]. Out of our 679 genes, 233 of them overlapped with their findings while 466 of our genes were unique.

### Sex and age on COPD expression

We found 9 statistically significant interacting genes between disease status at sex from our LRT results. These genes are BCORP1, EIF1AY, KDM5D, MAP7D2, PRKY, RPS4Y1, TTTY10, TTTY14, and USP9Y. The majority of these genes are Y-linked such as TTTY10 which is testes specific.

**Fig 10:**
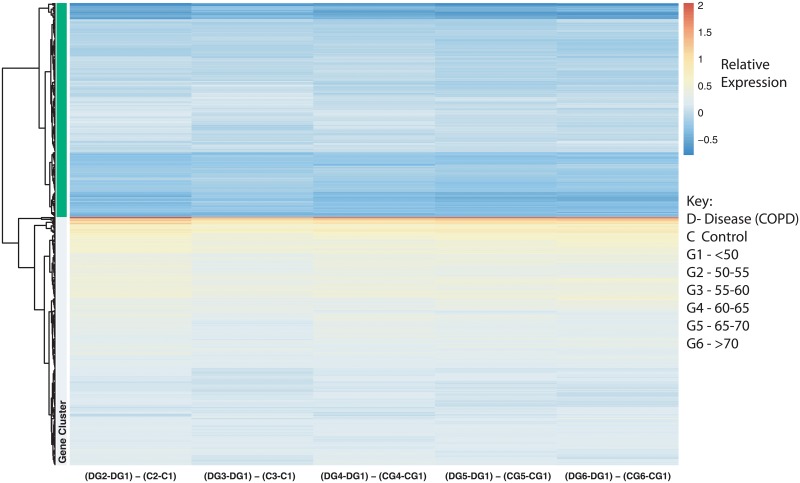
Heatmap of age effect on the statistically significant disease gene list (biologically significant). The enrichment analysis was based on the 679 statistically significant differentially expressed genes filtered for effect size. Comparison of baseline-adjusted estimates for control subjects and COPD subjects.

To determine the age effect on our DEG associated with COPD (679 genes), we focused on our LRT results where the age group <50 was the baseline. We plotted the relative expression (difference in estimates) across all age comparisons with <50 as the baseline by disease status. While the results are indicative of modest changes in two clusters, one showing higher expression in COPD in the >50 age groups, and one showing low expression in the >50 age groups, there is minimal observable variation between the age groups. (Fig 10). Furthermore, we did not find any statistically significant genes with an interaction between disease status and age from our LRT results.

### Machine learning with COPD data

**Fig 11:**
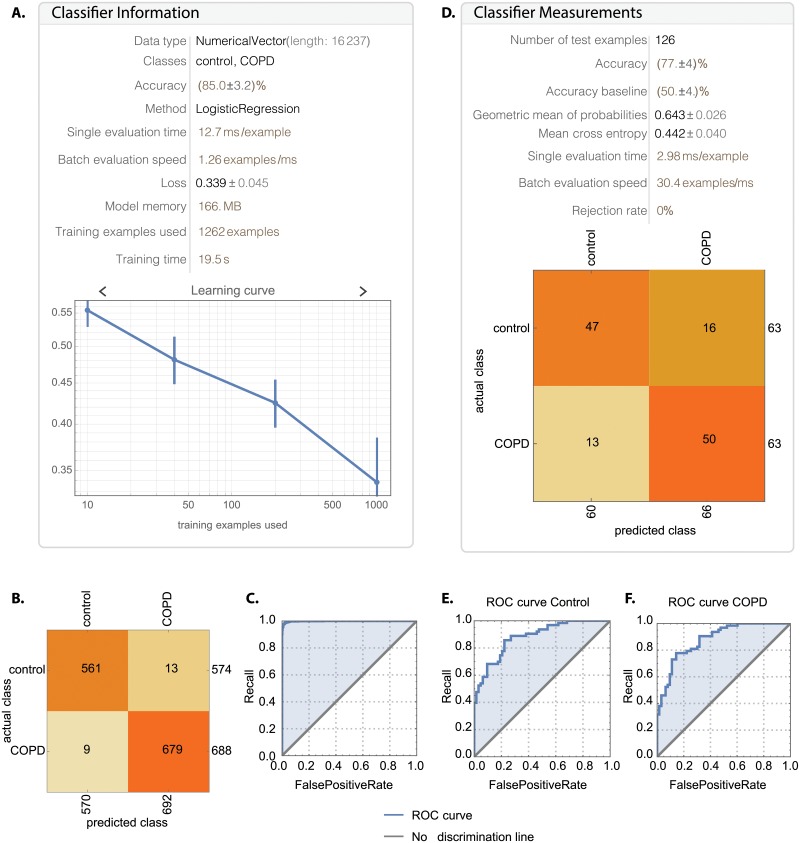
Trained logistic regression model can classify COPD and healthy profiles. (A)The logistic regression model trained on all the data achieves 85.0±3.2% accuracy), with the (B) confusion matrix and (C) ROC curves indicating good performance overall, with AUC 0.998. Training with 10-fold cross validation gives an average accuracy of 81.7±3.1%, with the worst testing model shown in (D) and its ROC for (E) Controls and (F) COPD shown respectively, with an AUC of 0.881.

Using the estimated gene expression from all common we trained a logistic regression model in Mathematica for predicting whether a profile belongs to the control or COPD group. Training with all samples achieved an accuracy of 85.0±3.2%, (Fig 11A). The corresponding confusion matrix and receiver operating characteristic (ROC) curves are shown in Fig 11B and 11C respectively, with an ROC area under the curve (AUC) of 0.998. Furthermore, we decided to carry out a 10-fold cross-validation analysis of randomized order samples, where we trained on 90% of the data each time and tested on the remaining 10%. On average the model had an accuracy of 81.7% (standard deviation of 3.1%), and ROC AUC of 0.910 (standard deviation of 0.021). An example of the worst performing realization from the cross-validation is shown in Fig 11D and 11F, where 47/63 controls and 50/63 COPD samples were classified correctly, whereas 16/63 controls samples were misclassified as COPD, and 13/63 COPD were misclassified as controls. Equivalently, the false positive rates were on average 0.20 (control) and 0.16 (COPD), and the false discovery rates were on average 0.22 (control) and 0.15 (COPD).

## Discussion

Chronic obstructive pulmonary disease causes damage to the lungs because of exposure to toxic irritants or genetic factors, and is a rising global health problem. With an increase in the elderly population’s life expectancy and the number of smokers, the prevalence of COPD and its morbidity rates are expected to rise. Researchers are working to identify strategies that can help to clearly understand COPD, its pathology, and to find biomarkers in easily accessible body fluids to promote earlier detection of COPD and improve accuracy of diagnosis [14–16]. Our research objective was to identify age, sex and smoking status effects on gene expression between COPD and controls in blood. We curated and downloaded 7 microarray expression datasets for our re-analysis on COPD. Using the raw expression data, we removed the background, annotated and summarized the probes, and merged the 7 datasets together by common gene names. This was followed by data normalization using BoxCox power transformation and downstream analyses to identify differentially expressed genes and genes that were biologically significant. This is the largest COPD reanalysis and explores expression variability in 1,262 samples by modeling linear and binary effects of disease status, age, sex and smoking status.

Our LRT highlighted 3,315 statistically significant (q-value <0.05; disease status factor) disease genes (see DF4 and DF6 of online supplementary data files). One of our top genes, PLD1, has previously been associated with COPD susceptibility [52]. Other genes such as GPR15 have also been associated with COPD and inflammation within the lungs. Our pathway enrichment results include Cytokine-cytokine receptor interactions and other immune related pathways (Table 3) and GO terms results include innate immune response, adaptive immune response and inflammation (DF10 of online supplementary data files) that have previously been associated with COPD. In the highlighted Cytokine-cytokine receptor interaction KEGG pathway there are different classes of cytokines such as chemokines, class I cytokines and the Tumor necrosis factor and Transforming growth factor beta families with varying expression (Fig 7). Cytokines play a major role in the inflammatory response observed in COPD subjects. For instance, CCR8 (chemokine) was up-regulated in COPD subjects (Fig 6). Increased levels of CCR8 has been previously observed in allergic asthmatics [53] and has a functional role in macrophage processes and release of cytokines in the lungs [54].

Additionally, we identified multiple genes associated with the Pathways in Cancer KEGG pathway (Fig 5). COPD is a known risk factor for lung cancer and it leads to 1% of cancer cases each year [55]. Furthermore, there is a five-fold increase to developing lung cancer in patients with COPD compared to individuals with normal pulmonary function [55]. Some of our highlighted genes are involved in apoptosis (Fas and CASP9), DNA damage (MDM2), Extra-cellular matrix (ECM) receptor interaction (ECM) and proliferation (CyclinD1) (Fig 5). We also visualized our up- and down- regulated gene hits in the other enriched KEGG pathways (Table 3) such as the Wnt signaling pathway (previously associated with the pathogenesis of COPD and causing inflammation) [56] (Fig 6) and Notch signaling pathway (Fig 8) which plays a role in lung development [57].

Focusing on the 679 differentially expressed disease genes (filtered for biological effect), some of the top up-regulated genes are DUSP7 (MAPK signaling), GPR15 (found on lymphocytes and involved in trafficking of lymphocytes), PLD1 (signal transduction), FICD (protein adenylyltransferase) and CBLL1 (proto-oncogene) [58] (Table 4). As for our top down-regulated genes RPS4Y1 (ribosomal protein), FCGR1B (role in immune response) and TCF7 (role in natural killer cell development) [58]. We also wanted to compare our gene list to a previously published meta-analysis. Reinhold et al., had a total of 6,243 genes which they grouped into 15 modules for each cohort. Out of our 679 genes, 233 of them overlapped with their findings while 466 of our genes were unique.

To assess the effect of smoking status on gene expression, we focused on the genes with a significant interaction between disease status and smoking status. We identified 24 disease genes that significantly interacted with smoking status (Fig 9). The baseline in Fig 9 was our non-smoking controls. For the control groups, current and former smokers display down-regulated expression in these select genes compared to non-smoking controls. There are a couple genes that are slightly elevated (LPAL2 and SFXN3). This indicates changes due solely to smoking with moderate differences between former and current smokers. As for the COPD smokers and non-smokers, the majority of these genes are elevated compared to non-smoking controls with the exception of RPL10L (ribosomal protein), RPS4Y1 (ribosomal protein), EIF1AY (translation initiation factor), (transcription factor), (histone binding protein) and DDX3Y (involved in transcription) being down-regulated in COPD compared to healthy non-smokers. Some of these genes have been associated with lung function and disorders such as DLEU7 which has been previously associated with lung function decline [59] (Fig 9). In our datasets there was only 1 COPD non-smoker which was excluded from this analysis.

As for sex specific effects on gene expression, we identified 9 disease genes with a statistically significant interaction with sex. Studies on COPD and sex, previously suggested higher prevalence in males due to them having higher smoking rates [60, 61]. However, currently with larger numbers of women smoking the prevalence of COPD in women is on the rise. Studies have shown that women are 50% more susceptible to COPD than males and why this is the case is still an on going debate [60, 61]. Some reasons include, smaller airways so larger concentrations of tobacco smoke in the lungs and hormonal effects [60, 61].

Aging trends were visualized on the 679 biologically significant disease genes. Symptoms for COPD can be detected between ages 40 and 50 [62], and because of this we used our subjects grouped as <50 as our baseline. The data clustered into two distinct groups: group 1 with genes showing lower expression in COPD in the >50 age groups, and group 2 showing higher expression in the >50 age groups, with minimal observable variation between the >50 age groups. (Fig 10). The genes in group 1 did not result in any statistically significant Reactome pathways. However they have been associated with the Neutrophil degranulation pathway, notch signaling, chemokine receptors, cancer pathways and transcription. The genes in group 2 have been previously associated with interleukin signaling pathways and calcium channels. We also did not identify enriched Reactome pathways for this gene list. In addition to this, we did not find any significant interacting genes between disease status and age.

To test the possibility of using blood expression data from micro-arrays to predict disease status, we performed machine learning with a logistic regression model using the full common array genes. This resulted in an average accuracy of 81.7% (Fig 11). These results are promising despite using aggregate expression versus cell-type specific expression. Previous studies explored using computed tomography (CT) images COPD patients and controls for disease classification [63]. Some studies also used patient reported data (such as heart rate, respiratory rate) to predict disease exacerbation and resulted in an ROC of 0.87 [64] and another with 70% sensitivity and 71% specificity [65].

Conducting a reanalysis with microarray expression data limits our findings to annotated genes, and hinders us from discovering novel genes and looking at the entire transcriptome. Additionally, using publicly available data limits us to specific factors we can explore in our analysis due to subject characteristics not being reported uniformly across datasets (see S2 File). For example, all studies did not report ethnicity and therefore we could not investigate the effect of ethnicity on gene expression in COPD. This would be a good factor to explore due to over 90% of COPD cases occurring in low-middle class communities [5, 10]. We also did not have consistently reported disease severity information to factor into our analysis and findings. Our selection criteria for the publicly available data limits our sample size (Fig 2). In addition to this, the limitations of available data resulted in unbalance in sample constitution: 1,262 samples with 574 controls and 688 COPD, of which 792 are males and 470 females, and have smoking status as 183 non-smokers, 418 smokers, and 661 former smokers. As for our machine learning algorithm, despite having a good predictive power and accuracy, we could not explore cell-type specific data. Furthermore, the observed confounding between studies suggests that samples would need to be analysed together with the current sample sets in new investigations, prior to prediction of status.

Our study highlights new gene candidates by factor (disease status, age, sex and smoking status) and genes that statistically interact between disease status and smoking status that can be studied further to understand their role in COPD. Future work to expand on our findings must include the use of cell-type specific expression data and RNA-sequencing data. Due to COPD being characterized by inflammation, increased macrophages and neutrophils and their release of cytokines, looking at cell-type specific data can give more insight on pathology of COPD. Using cell-type specific data for predicting disease states will also expand on our findings. RNA-sequencing data can introduce novel gene candidates and biomarkers for COPD. Furthermore, implementing proteomics and metabolomics can help characterize disease pathology and may lead to discovery of additional signatures for early detection of COPD using a systems biology approach.

## Supporting information

S1 File Preferred Reporting Items for Systematic Reviews and Meta-Analyses (PRISMA) checklist. The file lists the manuscript sections corresponding to the PRISMA reporting checklist requirements.
(DOC)

S2 File Datasets and the information reported on samples used for the re-analysis. This Microsoft Excel file lists all of the studies included in the re-analysis ans well as their sample description and study details. It highlights factors not commonly reported across all datasets.
(XLSX)

S3 File Description of our online supplementary data. This Microsoft Excel file lists all of our supplemental data files (datasets and results) from our re-analysis. See our data availability statement for more information.
(XLSX)
